# Clinical Organizational Science: an integrative framework for structural intervention in complex organizations

**DOI:** 10.3389/fpsyg.2026.1827324

**Published:** 2026-04-30

**Authors:** Makoto Yamanaka, Masaya Nakamori

**Affiliations:** DroR Corporation, Tokyo, Japan

**Keywords:** Clinical Organizational Science, complex adaptive systems, organizational change, organizational development, organizational intervention, organizational transformation, psychological safety, structural intervention

## Abstract

Organizations frequently fail to sustain transformation despite substantial investment in leadership development, cultural change programs, and structural redesign. Existing frameworks tend to treat organizational stability as passive inertia rather than as an actively and recursively reproduced dynamic state. This paper introduces Clinical Organizational Science (COS), an integrative framework that addresses this gap by specifying the structural mechanisms through which organizational stability is generated and through which durable transformation can be induced. Drawing on complexity science, neuroscience, organizational psychology, and behavioral science, COS proposes a multilevel mechanistic account—the emergence bridge—connecting individual behavioral habituation to organizational-level attractor transition. Three structured intervention techniques are presented—Field Gradient Theory, Loop Conversion Design, and Neural Base Design—each operationalizing a distinct structural mechanism with specified theoretical foundations and failure conditions. Illustrative patterns consistent with the framework’s theoretical predictions are discussed across behavioral, relational, and organizational levels, with explicit acknowledgment of their conceptual rather than evidentiary status. An ethics framework governing neuroscience-informed organizational intervention is also proposed. COS offers a theoretically grounded conceptual framework that repositions organizational transformation from a behavioral change project to a structural intervention problem, accompanied by testable propositions and directions for independent empirical investigation.

## 1 Introduction

### 1.1 The problem: why organizations resist change

A persistent paradox confronts practitioners and scholars of organizational change: organizations frequently resist transformation even when their members consciously desire it, even when leadership mandates it, and even when substantial resources are committed to enabling it. Although the precise magnitude of organizational change failure is methodologically difficult to establish—widely cited figures suggesting failure rates of 60–70% have been questioned on grounds of measurement rigor (Hughes, 2011; Burnes and Jackson, 2011)—the preponderance of evidence from multiple independent research traditions converges on the conclusion that large-scale organizational transformation consistently falls short of intended outcomes (Rafferty et al., 2013; Oreg et al., 2011; Worley and Mohrman, 2014). This persistent pattern is not merely a failure of execution. It is a signal that our theoretical understanding of why organizations maintain their current states is incomplete.

The dominant approaches to organizational change—leadership development, cultural intervention, structural redesign, and training programs—share a common underlying assumption: that organizations change when the people within them change their beliefs, attitudes, or behaviors. This assumption is not false, but it is insufficient. It treats organizational stability as the absence of sufficient impetus for change, rather than as an actively produced and maintained dynamic state. As long as stability is conceptualized as inertia, interventions will continue to target the symptoms—surface behaviors—without addressing the structures that generate and regenerate those behaviors.

This paper argues that organizational stability is better understood as the recursive reproduction of structural interaction patterns, and that effective organizational transformation therefore requires intervention at the level of those structures rather than at the level of behaviors or attitudes alone. This reframing is not merely theoretical: it has direct implications for how interventions are designed, implemented, and evaluated.

### 1.2 Existing approaches and their limitations

Organizational psychology and management science have produced rich bodies of knowledge about organizational change. The psychological safety literature (Edmondson, 1999) has established that interpersonal risk perception is a critical mediator of team learning behavior. Lewin's (1951) field theory articulated that behavior is a function of both the person and their environment—B = f(P, E)—establishing a conceptual foundation for structural intervention and inspiring a tradition of experiential and action-oriented intervention methods (Marrow, 1969). Routine theory (Feldman and Pentland, 2003) demonstrated that organizational patterns are not monolithic but are constituted through the dynamic interplay of ostensive and performative dimensions. Complex systems approaches (Stacey, 2010; Kauffman, 1993) have shown that organizations exhibit nonlinear, path-dependent dynamics that resist top–down control.

These contributions are substantial. Yet when practitioners attempt to translate them into intervention designs, a persistent gap remains. Psychological safety research identifies what to cultivate but provides limited guidance on how to structurally embed it through reproducible techniques. Lewin’s field theory, while generative as a conceptual framework, has not been systematically operationalized into a coherent set of structural intervention techniques applicable across organizational contexts. Routine theory describes the structure of organizational patterns but does not specify how to shift them. Complexity science captures the nonlinearity of organizational dynamics but offers few actionable intervention principles.

Two adjacent fields require differentiation. Organizational Neuroscience (ON) and Organizational Cognitive Neuroscience (OCN), as formulated by Becker and Cropanzano (2010) and Senior et al. (2011), apply neuroscientific measurement to investigate the neural substrates of organizational behavior. COS is not a contribution to this paradigm: it does not employ neural measurement and does not seek to establish neural correlates of organizational phenomena. The Neuroleadership field (Rock, 2010) draws on neuroscience to inform leadership development, but focuses on individual cognitive and emotional regulation rather than structural organizational dynamics. COS differs from both in three respects: (1) it integrates complexity science and attractor dynamics as its primary theoretical architecture, with neuroscience serving as a coherence layer rather than the dominant framework; (2) it targets structural intervention at the organizational system level rather than individual neural states; and (3) it operationalizes its account into reproducible intervention techniques with specified mechanisms and failure conditions. The ‘clinical’ framing refers to the practitioner’s embedded engagement with the organizational system, not to clinical neuroscience methodology.

What is needed is a framework that not only integrates these insights but operationalizes them as a coherent set of intervention techniques—techniques grounded in scientific theory yet applicable in the complex, context-dependent reality of organizational life.

### 1.3 Introducing Clinical Organizational Science

This paper presents Clinical Organizational Science (COS), an integrative applied framework that synthesizes complexity science, neuroscience, organizational psychology, and behavioral science into a coherent account of organizational stability and structural intervention.

The term ‘clinical’ is deliberate and carries specific theoretical weight. In medicine, clinical practice refers not to consultation from a distance but to intervention at the bedside: continuous presence within the system under observation, sustained engagement with its dynamics over time, and iterative refinement of intervention in response to observed effects. COS adopts this posture toward organizations. Rather than diagnosing from the outside and prescribing from above, COS practitioners embed within the organizational system—in DroR’s case, through a Business Process Outsourcing (BPO) arrangement—and intervene from within, continuously observing and adapting. This posture extends Schein’s (1999, 2001) tradition of process consultation and clinical inquiry, which established embedded, iterative practitioner engagement as the appropriate mode for organizational work; COS adds a structural intervention architecture specifying the mechanisms through which such engagement produces durable change.

The term ‘clinical’ also carries an epistemological commitment. Clinical medicine does not claim to fully predict or control the course of a disease; it claims to understand the mechanisms well enough to influence the probability of favorable outcomes. COS adopts the same posture: it does not claim to engineer specific organizational outcomes, but to understand organizational stability well enough to increase the probability of structural transition by designing conditions under which such transitions become more likely. We acknowledge that clinical medicine maintains an institutional separation between treatment and research roles that COS does not replicate—the same practitioners who design and deliver interventions also observe and report on their effects. This limitation is addressed directly in Section 4.

COS is advanced not as a completed empirical science but as a *mechanistic framework accompanied by testable propositions*. Three distinctions define its novelty: (1) not ‘psychological safety as a climate outcome to be cultivated’ but ‘psychological safety as a structural condition to be engineered’; (2) not ‘feedback as an interpersonal skill’ but ‘feedback as a cybernetic architecture subject to deliberate redesign’; (3) not ‘organizational change as a behavior shift’ but ‘organizational change as attractor transition induced through reinforcement pathway perturbation.’ These distinctions generate different intervention designs and different empirical predictions, and constitute the theoretical contribution this paper advances.

### 1.4 Structure of this paper

The remainder of this paper is organized as follows. Section 2 presents the theoretical foundations of Clinical Organizational Science, defines its core concepts, situates it within existing literature, and specifies how it advances beyond existing frameworks. A note on ethical governance principles concludes Section 2, with full elaboration in Section 5. Section 3 describes the three intervention techniques that constitute the operational core of COS: Field Gradient Theory, Loop Conversion Design, and Neural Base Design. Section 4 presents conceptual illustrations derived from the framework’s structural predictions, accompanied by explicit caveats on their conceptual—rather than evidentiary—status. Section 5 addresses the ethical governance framework that COS requires, given its integration of neuroscience into organizational intervention. Section 6 discusses theoretical and practical implications, limitations, directions for future research, and concludes.

## 2 Theoretical foundations of Clinical Organizational Science

### 2.1 Defining Clinical Organizational Science

Clinical Organizational Science is defined as a mechanistic framework for organizational intervention that integrates four scientific domains—complexity science, neuroscience, organizational psychology, and behavioral science—to design, implement, and evaluate structural interventions in complex organizations. COS conceptualizes organizations as multilevel complex adaptive systems in which stability emerges from the recursive reinforcement of interactional patterns, and in which transformation requires perturbation at the level of those reinforcement structures rather than modification of surface behaviors.

Three foundational concepts require explicit definition to prevent misreading throughout this paper.

An *attractor*, as employed in COS, refers to a stable pattern within an organization’s behavioral state space toward which the system tends to return following perturbation. This usage draws on complexity adaptive systems theory (Kauffman, 1993; Stacey, 2010) but applies it to organizational phenomena: the state variables in question are not physical quantities but organizational observables such as communication response patterns, decision-making protocols, norms of emotional expression, and habitual patterns of collaborative behavior. An attractor in this sense is an organizational tendency—a default mode that reasserts itself when external forces relax. This usage is an organizational adaptation of the complexity science concept, not a claim of strict physical equivalence.

*Perturbation*, as used in COS, refers to an intentional structural change that moves the organizational system away from its current attractor state by altering the conditions that sustain that state. Critically, perturbation in the COS framework is not imposed from outside the system; rather, it is induced from within the system through structural intervention in interaction patterns, feedback loop architecture, and habitual behavioral sequences. This distinction—between external pressure and internal structural reconfiguration—is central to the COS approach and reflects Stacey’s (2010) analysis of the limits of top–down organizational control.

*Neural Base Design*, the third technique to be described in Section 3, is defined here to prevent a common misreading: it does not involve direct measurement or manipulation of neural activity. No neuroimaging or neurostimulation is employed. Rather, it refers to the structural design of behavioral practices—habitualization, gratitude sharing, somatic awareness check-ins—that are informed by neurological theory regarding habit formation and social bonding. Neuroscience theory serves as an explanatory framework, not as a basis for direct neural intervention.

*Operational indicators of attractor state*. Because ‘attractor’ is a theoretical construct, the question of what practitioners actually observe when assessing an organization’s attractor state requires explicit treatment. COS identifies three observable categories that, taken together, characterize an organizational attractor: (1) *Communication latency and acknowledgment norms*—the typical elapsed time between message receipt and acknowledgment, and whether acknowledgment occurs spontaneously or only under explicit prompting; (2) *Voice distribution in group settings*—the pattern of who speaks, who is listened to, and who self-censors across hierarchical levels and in the presence of senior members; (3) *Negative information disclosure response*—whether the sharing of errors, failures, or problematic data is met with defensive dynamics or received as an act of organizational trust. These categories are not exhaustive but represent candidate observational markers of attractor state that COS proposes for future empirical validation. A shift in these indicators is predicted to constitute a primary signal of attractor transition.

### 2.2 The four scientific domains

COS integrates four scientific domains, each contributing a distinct but complementary explanatory layer.

*Complexity science* provides the macro-level framework for understanding organizational dynamics. Drawing on Kauffman’s (1993) analysis of complex adaptive systems and Stacey’s (2010) application of complexity theory to organizational life, COS treats organizations as nonlinear, path-dependent systems in which small differences in initial conditions can produce large divergences in outcomes, and in which causal relationships are often identifiable only retrospectively. Prigogine and Stengers’s (1984) dissipative structures theory contributes the insight that transitions between stable states require the introduction of a gradient—a directed differential of energy or influence—that enables the system to move from one equilibrium basin to another. von Bertalanffy’s (1968) general systems theory provides the theoretical warrant for applying insights across physical, biological, and social systems, grounding the cross-domain integration that COS requires.

*Neuroscience* provides the micro-level foundation for understanding why individuals behave as they do within organizational systems, and why structural change at the organizational level may require changes in the neural substrates of individual habit. The neuroscientific layer in COS does not function as an independent explanatory necessity; rather, it serves as a theoretical inspiration and coherence layer, aligning behavioral intervention design with established findings on habit consolidation and affective regulation at the individual level. Kandel’s (2006) foundational work on synaptic plasticity establishes that repeated behavioral patterns structurally alter neural connections—that experience physically rewrites the brain—providing the theoretical basis for the COS prediction that sustainable organizational change requires habitualization of new behavioral patterns rather than one-time instruction. Research on social bonding (Uvnas-Moberg, 1998; Algoe et al., 2010) informs practices intended to promote trust and affiliation. Damasio's (1994) somatic marker hypothesis motivates somatic awareness practices in Neural Base Design. The dopaminergic reward prediction literature (Schultz et al., 1997) informs the design of motivational structures intended to sustain participation in organizational rhythms. Throughout this paper, neuroscientific frameworks serve as theoretical inspiration informing behavioral intervention design; claims about specific neural mechanisms are theoretical predictions, not empirically verified accounts of what is occurring in the neural systems of organizational members.

*Organizational psychology* provides the meso-level concepts for understanding how individual psychology aggregates into collective organizational phenomena. Lewin’s (1951) field theory—B = f(P, E)—establishes that behavior is constituted by the interaction of individual dispositions and environmental conditions, grounding the COS commitment to environmental structural design as the primary mode of intervention. Edmondson’s (1999, 2018) research on psychological safety identifies interpersonal risk perception as a critical regulator of learning and performance behavior in teams. Weick’s (1995) sensemaking theory articulates how organizational members collectively construct meaning from ambiguous experience, a process that COS treats as a mechanism for stabilizing new attractor states once perturbation has occurred.

*Behavioral science* provides intervention design principles at the level of individual behavior and habit formation. Fogg’s (2019) behavior design framework—particularly the concept of ‘tiny habits’ anchored to existing behavioral sequences—informs the design of the periodic organizational rhythms that Neural Base Design employs. Wiener’s (1948) cybernetic analysis of feedback mechanisms, particularly the stabilizing function of negative feedback loops, provides the engineering logic for Loop Conversion Design. Meadows’s (2008) identification of feedback loop structure as a high-leverage intervention point in complex systems grounds the theoretical claim that structural feedback redesign is more effective than behavioral persuasion.

### 2.3 Organizations as complex systems of complex systems

A distinctive feature of the COS framework is its explicit attention to hierarchical complexity. The human brain is itself a complex system, giving rise to emergent phenomena that cannot be reduced to individual neurons. When multiple brains interact within an organizational context, the resulting collective system is a complex system composed of complex systems—a second-order complexity whose dynamics cannot be predicted from individual-level analysis alone.

This hierarchical structure has direct implications for intervention design. Interventions targeting individual-level variables may be effective at that level while producing little organizational change, because the organizational attractor persists through the mutual reinforcement of interactional patterns even when individual components have changed. Conversely, structural changes at the organizational level—in communication protocols, meeting designs, or feedback architectures—can produce individual-level changes through the environmental pressures they generate, consistent with Lewin’s field theory.

Feldman and Pentland’s (2003) organizational routines theory provides a theoretically precise account of this inter-level relationship: organizational routines are stable patterns that emerge from repeated interactions, not from any single individual’s behavior. Neural Base Design exploits this insight by systematically altering the behavioral inputs individuals bring to organizational interactions, seeking to alter the conditions under which routines are reproduced. Stacey’s (2010) complexity theory cautions that neither direction nor content of emergent change can be specified in advance—intervention can increase the probability of transition without determining its destination, an epistemological constraint the COS framework treats as a feature rather than a weakness.

### 2.4 Positioning COS within and beyond existing literature

COS does not claim to replace existing theoretical traditions but to extend and operationalize them. Table 1 presents the relationship between COS and the major theoretical frameworks it engages.

**Table 1:** COS and existing theoretical traditions.

Theoretical tradition	Shared insight	COS contribution
Structuration theory (Giddens, 1984)	Recursive reproduction of structure through agency	Specification of intervention techniques targeting reinforcement pathways
Organizational routines (Feldman and Pentland, 2003)	Emergent stability from repeated interaction	Neuro-informed theoretical account linking individual habit to organizational routine
Complexity theory (Stacey, 2010; Kauffman, 1993)	Nonlinear, attractor-based organizational dynamics	Operationalization into three structural intervention techniques
Psychological safety (Edmondson, 1999)	Interpersonal safety as condition for organizational learning	Repositioned as structural attractor condition, engineered through specific practices
Action research (Lewin, 1946; Argyris and Schön, 1978)	Practice-theory iteration as knowledge generation	Conceptual illustration of predicted patterns across organizational contexts

The most significant advance COS offers beyond structuration theory (Giddens, 1984) is the specification of intervention mechanisms: Giddens articulates the recursive reproduction of structure but does not specify how this recursion can be interrupted. The advance beyond routine theory (Feldman and Pentland, 2003) is the integration of neuroscience as a theoretical explanatory layer connecting individual habit to organizational routine reproduction. The advance beyond psychological safety research (Edmondson, 1999) is the reframing of psychological safety from a cultural outcome to a structural condition to be engineered through specific, reproducible practices.

#### 2.4.1 Positioning COS relative to implementation science and organizational readiness

COS also addresses challenges—adoption, sustainability, behavioral embedding, and system-level change—that overlap with the concerns of implementation science. It is useful to clarify its relationship to established frameworks in this space.

The Consolidated Framework for Implementation Research (CFIR; Damschroder et al., 2022) provides a comprehensive taxonomy of implementation determinants across innovation, setting, individual, and process domains. CFIR offers diagnostic structure for identifying barriers and facilitators but does not specify the structural mechanisms through which intervention effects propagate across organizational levels or how new behavioral patterns become self-sustaining. COS operates at a different level of explanation: proposing how individual habituation aggregates into organizational attractor transition through the emergence bridge.

Integrative frameworks combining complexity science, implementation science, and social-behavioral dimensions have advanced this space further. PROLIFERATE (Pinero de Plaza et al., 2023) integrates ecological, mechanistic, and social logics to evaluate innovations within complex adaptive health systems; PROLIFERATE_AI (Pinero de Plaza et al., 2025) extends this through predictive and decision-support capabilities. These frameworks demonstrate the growing convergence between complexity thinking and implementation measurement. COS differs in primary orientation: whereas PROLIFERATE focuses on evaluating implementation products and processes, COS specifies the structural intervention mechanisms—influence gradients, feedback loop conversion, habitualized rhythms—through which organizational attractor states may be shifted. The frameworks are complementary rather than competing.

Weiner’s (2009) organizational readiness for change theory, conceptualizing readiness as shared commitment and collective efficacy, provides additional theoretical grounding. The conditions Neural Base Design seeks to create—psychological safety, trust, habitualized collaborative practices—can be understood as structural preconditions for the collective commitment and efficacy Weiner describes. COS extends this by specifying the behavioral mechanisms through which these shared psychological states may be produced and maintained.

Two adjacent practice traditions warrant explicit differentiation. Appreciative Inquiry (AI; Cooperrider and Whitney, 2005) shares with COS an emphasis on positive observation as an intervention lever. The differentiation lies in the explanatory framework: AI treats positive framing as a generative epistemological stance, whereas COS treats the structural preponderance of positive observations as a feedback architecture intervention with specified cybernetic properties—the conversion of a self-amplifying positive feedback loop into a self-correcting negative feedback system. This distinction generates different empirical predictions and different intervention designs. Somatic leadership approaches (cf. Strozzi-Heckler, 2014) similarly share an interest in body-state awareness, but COS’s somatic axis is distinguished by its integration within a broader multilevel framework connecting individual somatic awareness to organizational attractor dynamics, rather than treating somatic practice as a standalone modality.

### 2.5 A note on ethical governance

Because COS employs neuroscience language to describe organizational intervention, it is necessary to address potential ethical misreadings before proceeding to the technique descriptions in Section 3. COS does not involve direct manipulation of neural states, covert influence, or any practice inconsistent with the autonomy and dignity of organizational members. All COS interventions operate on the principles of transparency (explicit disclosure of purpose and method), participation (collaborative design with client organizations), autonomy (no practice compels behavior against individual will), and revocability (any intervention structure may be withdrawn at participant request). These principles are elaborated fully in Section 5. Readers are invited to hold this commitment in mind as the intervention techniques are described in the following section.

**Figure 1:**
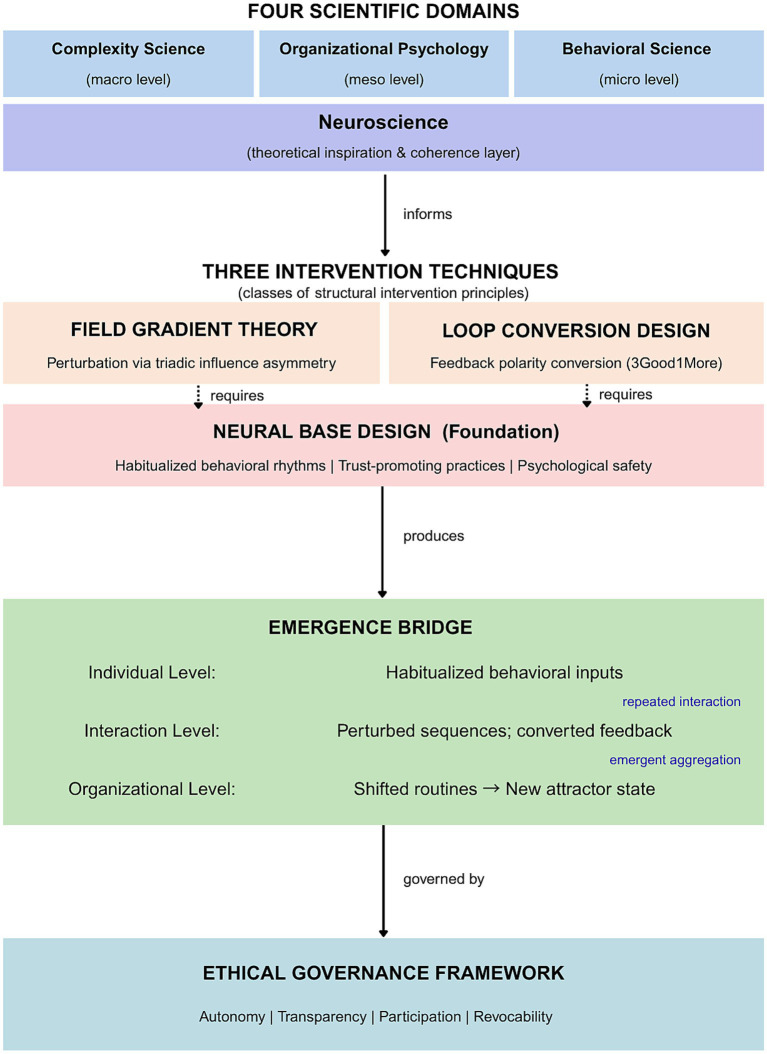
Integrative conceptual overview of Clinical Organizational Science: four scientific domains, three intervention techniques, the emergence bridge mechanism, and ethical governance framework.

Figure 1 provides an integrative overview of the COS framework, illustrating the relationships among its four scientific domains, three intervention techniques, the emergence bridge mechanism, and ethical governance principles.

## 3 The three intervention techniques

### 3.1 Overview: a hierarchical architecture

The three intervention techniques of COS represent classes of structural intervention principles, organized in a hierarchical architecture in which each creates conditions for the others to function effectively. While described here through specific implementations developed in practice, the underlying principles are generalizable design constructs that can be independently adapted and tested across organizational contexts. Neural Base Design forms the foundation: it builds the neurological and relational conditions—habitualized behavioral rhythms, trust-promoting interaction practices, psychological safety—without which the other two techniques cannot sustain their effects. Field Gradient Theory and Loop Conversion Design operate above this foundation: Field Gradient Theory introduces structural asymmetries that enable perturbation of existing attractor states, while Loop Conversion Design converts destabilizing positive feedback dynamics into stabilizing negative feedback dynamics, preventing the system from collapsing into disorder during the transition between attractors.

**Figure 2:**
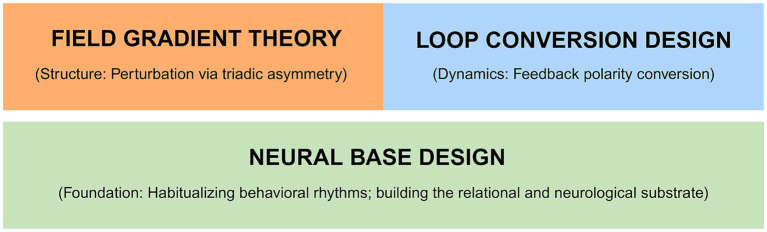
The hierarchical architecture of COS intervention techniques.

Figure 2 schematizes this architecture.

This architecture has a practical implication: interventions that deploy Field Gradient Theory or Loop Conversion Design without the foundational conditions created by Neural Base Design are unlikely to produce durable effects. The attractor-shifting dynamics of Field Gradient Theory require psychological safety to function as intended rather than as coercive pressure. The feedback conversion of Loop Conversion Design requires a baseline of positive relational experience—maintained by Neural Base Design—to make the identification of positive observations cognitively accessible.

### 3.2 Technique 1: field gradient theory

#### 3.2.1 Theoretical basis

Field Gradient Theory draws on four converging theoretical sources. Lewin’s (1951) field theory establishes that behavior is a function of the total psychological field in which a person is embedded—the constellation of forces, valences, and vectors that constitute their life space at any moment. From this foundation, Field Gradient Theory derives the principle that changing behavior requires changing the field in which behavior occurs, not only the dispositions of the individual actor.

Simmel’s (1908) sociological analysis of the triad provides the structural basis for the theory’s core mechanism. Simmel argued that the dyad and the triad are qualitatively different social forms: the dyad tends toward symmetry and equilibrium, with each party’s influence constrained and balanced by the other’s; the triad introduces an irreducible asymmetry, as any configuration of three parties generates coalitions, mediations, and influence differentials unavailable in a two-party structure. This structural asymmetry is, in Field Gradient Theory’s terms, a gradient—a directed differential in influence that can drive system movement.

Kauffman’s (1993) attractor concept provides the dynamical systems framework within which this gradient operates. An organizational system in an attractor state tends to resist perturbation and return to that state following disturbance. To produce lasting change, perturbation must exceed the attractor basin’s restoring force—it must push the system beyond the threshold at which a new attractor becomes more probable than the existing one.

#### 3.2.2 Core mechanism

The core mechanism of Field Gradient Theory is the deliberate design of triadic interaction structures—what we term the 2-on-1 configuration—that introduce asymmetric influence dynamics into organizational communication. In a 2-on-1 configuration, two parties direct their attention, engagement, and communicative energy toward a third party. This creates an influence gradient: the third party experiences a differential of social force that the existing attractor cannot absorb without disturbance. The gradient functions as a perturbation in the complexity science sense—a structural condition that increases the probability of attractor transition. It should be noted that Simmel’s (1908) original analysis concerned triadic dynamics as they arise organically in social life; whether intentionally designed meeting formats produce structural effects equivalent to those spontaneous triadic configurations is itself an empirical question that the present framework treats as a theoretically motivated working hypothesis rather than an established equivalence.

It is important to distinguish this mechanism from social pressure or coercion. The gradient produced by a 2-on-1 configuration is not a command or a demand; it is a change in the social field that makes certain behavioral responses more probable and others less probable, consistent with Lewin’s field-theoretic logic. Whether the third party moves in the direction the gradient suggests depends on many factors, including the content of the interaction and the relational conditions in which it occurs. Field Gradient Theory claims only to increase the probability of movement, not to determine its direction or magnitude.

#### 3.2.3 Practical implementation and failure conditions

The standard implementation of Field Gradient Theory is the 2-on-1 meeting structure, in which two individuals—typically including a DroR consultant or a trained internal facilitator—conduct a meeting with a third party in which the triadic influence configuration is intentional rather than incidental. The 3-on-1 configuration is employed in cases requiring stronger perturbation, where the organizational attractor is particularly deep or resistant to 2-on-1 dynamics.

*Known failure condition*: Field Gradient Theory produces its intended perturbation effect only when the relational conditions established by Neural Base Design are in place. When psychological safety (Edmondson, 1999) is absent, the 2-on-1 configuration does not function as an influence gradient—it functions as perceived coercive pressure, activating threat-related responses that trigger defensive behavior and reinforce rather than disrupt the existing attractor. This is not a marginal risk; it is a theoretically predictable failure mode that follows directly from the framework’s structural logic. The structural implication is that Field Gradient Theory deployed without Neural Base Design foundations is not merely ineffective but actively counterproductive, potentially deepening the attractor it is intended to disrupt. This failure condition is the structural rationale for the hierarchical architecture depicted in Figure 2 and for treating Neural Base Design as a prerequisite rather than a parallel component.

### 3.3 Technique 2: Loop Conversion Design

#### 3.3.1 Theoretical basis

Loop Conversion Design is grounded in cybernetics (Wiener, 1948) and systems thinking (Meadows, 2008). A terminological note: throughout this section, ‘positive’ feedback refers to self-amplifying loops that escalate deviation, and ‘negative’ feedback refers to self-correcting loops that restore stability—usage orthogonal to evaluative connotations. Meadows (2008) identified feedback loop structure as among the highest-leverage intervention points in complex systems.

The problem Loop Conversion Design addresses is the tendency of organizational social systems to generate self-amplifying positive feedback loops around negative content. Because human attentional systems exhibit a robust negativity bias (Baumeister et al., 2001), organizational communication in the absence of structural intervention tends to amplify criticism and interpersonal friction into a self-sustaining attractor state difficult to exit through behavioral persuasion alone. This structural problem cannot be solved by improving individual feedback skill: Kluger and DeNisi’s (1996) meta-analysis demonstrated that a substantial proportion of feedback interventions produce performance decrements, because effects depend on where attention is directed rather than on information content. Loop Conversion Design intervenes at the structural level—redesigning the feedback architecture rather than the skill of the giver.

Fredrickson’s (2001) Broaden-and-Build Theory provides a theoretical cognitive mechanism: positive emotional states expand individuals’ thought-action repertoires, suggesting that structurally requiring positive observations before developmental feedback may widen cognitive aperture and make recipients more capable of processing developmental feedback constructively. Specific ratio-based claims associated with this research have been subject to methodological criticism (Brown et al., 2013); the present application draws only on the core framework concerning cognitive broadening effects of positive affect.

#### 3.3.2 Core mechanism: 3Good1More

The operational implementation of Loop Conversion Design is the 3Good1More protocol: any critical or developmental feedback (designated ‘More’) is permissible only after three genuine positive observations (‘Good’) have been articulated. This structural rule converts the feedback interaction from a potentially runaway positive feedback loop—in which criticism begets defensiveness begets more criticism—into a bounded, negative feedback system in which developmental communication is embedded within a predominant context of affirmation.

The number three is not theoretically derived from first principles; it represents a practical threshold balancing genuine attentional engagement with sustainable habit formation. One positive observation risks being performed perfunctorily; five or more create cognitive load that may impede habituation. Three represents a working default that maintains the structural principle—positive observations must structurally exceed developmental ones—within realistic cognitive constraints. This value may be adjusted contextually (2:1 or 4:1 ratios may be appropriate in specific contexts), and the optimal ratio is itself a testable empirical question.

#### 3.3.3 Practical implementation

3Good1More is embedded as a structural protocol in the organizational rhythms designed by Neural Base Design: in weekly team meetings, retrospective sessions, and any formal feedback interaction. Its effectiveness depends on structural embedding—its function is not as a technique that individuals apply when they remember to do so, but as a design constraint that regulates interaction regardless of individual inclination or mood state.

### 3.4 Technique 3: Neural Base design

#### 3.4.1 Theoretical basis

Neural Base Design integrates multiple theoretical sources reflecting its foundational function. Kandel’s (2006) neuroscience of synaptic plasticity establishes that behavioral repetition physically rewrites the brain—synaptic connections mediating frequently-activated sequences are strengthened, reducing the cognitive effort required to initiate them. This provides the theoretical basis for the COS prediction that behavioral habits, once established, may become self-sustaining. Fogg’s (2019) behavior design framework provides the practical formation technology: new behaviors are most reliably habituated when anchored to existing behavioral sequences, rather than introduced as standalone practices.

Gratitude-sharing practices are informed by research demonstrating that gratitude expression reinforces social bonds through signals of attention and appreciation (Algoe et al., 2010; Colquitt et al., 2013). The original research context involves close interpersonal relationships—a difference from organizational settings that warrants acknowledgment—but the bond-reinforcing mechanism is not assumed to be relationship-type specific. Neurobiological accounts of social bonding (Uvnas-Moberg, 1998) are consistent with these behavioral findings, though specific neurobiological pathways in organizational settings have not been directly measured.

Damasio’s (1994) somatic marker hypothesis provides the basis for somatic check-in practices: decision-making is guided by body-state signals representing prior emotional experience, making structured somatic awareness a lever for earlier detection of stress or relational tension. Dopaminergic reward prediction theory (Schultz et al., 1997) informs motivational rhythm design: anticipatory activation toward reward-associated behaviors suggests that predictable positive reinforcement schedules may sustain participatory motivation without reliance on external enforcement. All neuroscientific claims in this section are theoretical predictions, not directly measured organizational outcomes.

#### 3.4.2 The four axes

Neural Base Design is structured around four axes: the *plasticity axis* (habit formation through repetition and anchor-based design; Kandel, 2006; Fogg, 2019); the *affiliative bonding axis* (interpersonal trust through structured gratitude expression; Algoe et al., 2010; Uvnas-Moberg, 1998—the term ‘affiliative bonding’ is preferred over narrower neurobiological labels to avoid overstating neurobiological specificity); the *motivational sustenance axis* (intrinsic motivational continuity through predictable reward structures; Schultz et al., 1997); and the *somatic awareness axis* (attentional awareness of body-state signals through structured check-ins; Damasio, 1994; Weick, 1995).

#### 3.4.3 Practical implementation: periodic rhythm design

Neural Base Design is operationalized as a system of daily, weekly, and monthly organizational rhythms, each targeting one or more of the four axes. The *daily rhythm* (brief morning stand-up) incorporates gratitude sharing, somatic check-in, and intention setting, maintaining the affiliative bonding conditions that support psychological safety throughout the working day. The *weekly rhythm* (structured team review) incorporates the 3Good1More protocol, sensemaking discussion, and review of interaction patterns, reinforcing the feedback architecture established by Loop Conversion Design. The *monthly rhythm* (extended retrospective) incorporates collective sensemaking around organizational priorities and somatic awareness practices to detect accumulated stress states, and serves as a review point for the overall COS intervention architecture.

#### 3.4.4 The emergence bridge: from individual habit to organizational attractor

A critical theoretical claim of COS is that changes in individual behavioral tendencies—produced through habitualization—aggregate through repeated interaction into changes in organizational-level stable patterns. This aggregation is emergent, not additive. Feldman and Pentland’s (2003) organizational routines theory provides the theoretical bridge: routines are altered when the behavioral inputs that participants bring to interactions change systematically. Neural Base Design produces this systematic change—in habitualized practices of gratitude expression, somatic awareness, and structured feedback—that feeds into the repeated interaction sequences through which routines are reproduced. As changed inputs become the normal behavioral environment, the routines that emerge shift, constituting a new organizational attractor that self-reproduces through participants’ mutual reinforcement.

**Figure 3:**
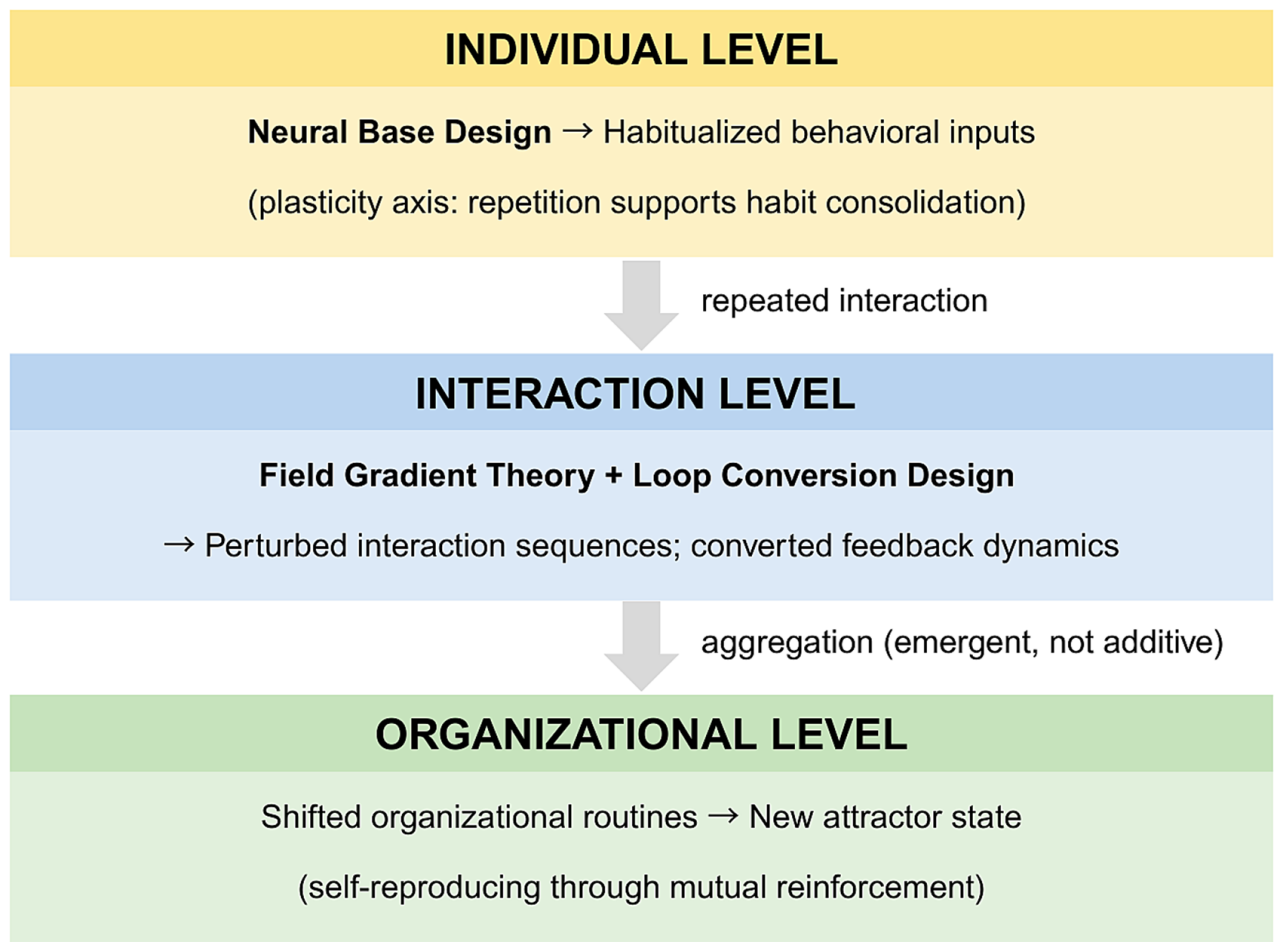
The emergence bridge: mechanism connecting individual habit to organizational attractor transition.

Figure 3 schematizes the emergence bridge mechanism.

## 4 Conceptual illustrations

### 4.1 Epistemological status of this section

This section presents conceptual illustrations to support the theoretical argument developed in Sections 2 and 3—not as empirical data, research findings, or systematic observational records. No original research data were generated or analyzed. These illustrations are theoretically consistent with the framework’s predictions; they are not intended to constitute evidence in the research sense, and no causal attribution is made. DroR practitioners serve simultaneously as framework designers, intervention deliverers, and authors, creating structural conditions for confirmation bias (Nickerson, 1998) and selective recall. This section should be read as conceptual illustration, not independent verification.

### 4.2 Illustrative patterns by level of analysis

The illustrative patterns below are theoretically derived from COS’s structural predictions and are consistent with the framework’s account of attractor dynamics. They are not drawn from a systematic dataset; they represent archetypal organizational configurations that the framework’s theory implies.

#### 4.2.1 Layer 1: behavioral and communication patterns

If Neural Base Design is sustained over time, COS predicts a qualitative shift in communicative responsiveness: rather than responding only when explicitly required, organizational members begin initiating acknowledgment of received messages and indicating expected response times before a fuller response is available. This qualitative shift—from reactive to proactively responsive communication—is theoretically consistent with the COS account of habitualized behavioral practices creating self-sustaining attractor states, and with Edmondson’s (1999) characterization of psychological safety norms.

#### 4.2.2 Layer 2: relational and psychological safety patterns

A second illustrative prediction concerns voice distribution in group settings. Under a hierarchical communication attractor—in which role-determined influence asymmetries concentrate meeting contributions among senior or high-status members—COS predicts that sustained Field Gradient Theory implementation will gradually shift participation toward broader distribution, including contributions from early-career members, as structural perturbation accumulates into a new interactional attractor. This is consistent with the emergent, non-deterministic character of attractor transition: a practitioner-level member begins making substantive contributions after several months—an outcome that cannot be engineered directly but becomes more probable under the structural conditions COS designs.

A third prediction concerns the reception of negative information. Under a threat-amplifying attractor, sharing problematic news worsens the relational tone of the interaction. Under sustained structural perturbation via Loop Conversion Design, COS predicts that negative information begins to be received as an act of organizational trust rather than a relational threat—a shift from a ‘threat’ attractor toward a ‘shared problem’ attractor, consistent with the technique’s theoretical mechanism.

#### 4.2.3 Layer 3: organizational pattern and emergence

At the organizational level, COS predicts that over approximately 6 months—proposed as a provisional temporal hypothesis—gratitude expressions begin circulating across hierarchical and departmental boundaries without practitioner facilitation: the predicted self-sustaining phase of attractor transition. This is theoretically consistent with Fogg’s (2019) habit consolidation timelines, Feldman and Pentland’s (2003) analysis of routine emergence, and Lally et al.’s (2010) finding that habit automaticity follows a gradual asymptotic trajectory with substantial individual variation.

### 4.3 Temporal structure: a testable prediction

A temporal pattern consistent with COS predictions warrants explicit statement as a testable hypothesis. The initial phase of COS implementation is characterized by a *volitional phase*: new practices are established within organizational rhythms, but participation remains dependent on active facilitation, consistent with Fogg’s (2019) analysis that new behaviors require sustained attentional resources until habit consolidation occurs.

Following a period of sustained implementation—on the order of months, with approximately 6 months proposed as a provisional temporal hypothesis—COS predicts a transition to an *autonomous phase*: participation becomes self-sustaining without practitioner prompting, and new organizational members adopt the practices as part of socialization into organizational norms. This prediction is consistent with Feldman and Pentland’s (2003) account of new organizational routines emerging from aggregated habitualized individual inputs, and with Lally et al.’s (2010) finding that habit automaticity follows a gradual asymptotic trajectory. This temporal prediction is explicitly offered as a testable hypothesis for future independent research, not as an established empirical finding.

## 5 Ethical governance framework

### 5.1 The necessity of an ethics framework

The integration of neuroscience concepts into organizational intervention creates two ethical risks: the misreading of scope (the impression that intervention directly manipulates neural states) and the misreading of intent (the impression that neuroscientific framing is employed to optimize covert manipulation). COS rejects both misreadings and constructs an explicit governance framework to prevent them.

### 5.2 Core ethical principles

The ethical governance framework of COS is constituted by four principles that govern the design and implementation of all interventions.

*Autonomy* is the foundational principle: COS treats the autonomy and dignity of each individual organizational member as an inviolable constraint on intervention design. No COS intervention is designed to circumvent, undermine, or exploit individual autonomy. Interventions are designed to create conditions that make certain choices more available or more probable, not to compel specific behaviors.

*Transparency* is the operational principle: the purposes, methods, and expected effects of COS intervention are communicated explicitly to client organizations and, where relevant, to organizational members. The use of neuroscience concepts in intervention design is disclosed, including the distinction between theoretical explanatory frameworks and direct neural intervention. This principle is analogous to the informed consent requirement in clinical medical practice—the ‘clinical’ metaphor that names this framework implies the ethical as well as the methodological posture of clinical medicine.

*Participation* is the collaborative principle: COS interventions are conducted with client organizations, not on them. The design, implementation, and evaluation of intervention architecture involves ongoing collaboration with organizational leadership and, as appropriate, with organizational members. This principle reflects COS’s commitment to practitioner-organization collaboration rather than expert-prescriptive delivery.

*Revocability* is the accountability principle: any COS intervention structure may be withdrawn or modified at the request of the client organization or of individual organizational members. No intervention is designed in ways that create dependency or make exit difficult. This principle is a structural safeguard against the use of COS techniques as instruments of organizational control rather than organizational development.

### 5.3 The structural vs. direct intervention distinction

COS intervenes in the behavioral and social conditions within which neural processes operate—interaction structures, feedback architectures, and habitualized practices—rather than in neural states directly. Direct intervention in neural states through pharmacological, electromagnetic, or other means would require a fundamentally different ethical framework, and is explicitly not what COS practitioners do. This is structural intervention, analogous to designing physical environments for health outcomes (urban planning for walkability) rather than direct medical treatment. Neuroscience theory in COS serves as an explanatory framework, not as a technology of neural manipulation—a distinction that must be communicated explicitly to client organizations as a component of the transparency principle.

## 6 Discussion

### 6.1 Theoretical contributions

This paper makes four theoretical contributions. First, Clinical Organizational Science is introduced as a named, integrative conceptual framework for structural organizational intervention—establishing a reference point for future theoretical development and empirical investigation.

Second, COS proposes an integrative theoretical account—the *emergence bridge*—connecting individual behavioral habituation to organizational-level attractor transition by synthesizing Kandel’s (2006) neural plasticity research, Feldman and Pentland’s (2003) organizational routines theory, and Kauffman’s (1993) attractor dynamics. This account is offered as a theoretically grounded hypothesis awaiting empirical verification, not as an established causal mechanism.

Third, COS repositions psychological safety (Edmondson, 1999) from a cultural outcome to be cultivated to a structural condition to be engineered—specifying the behavioral practices through which psychological safety is created and maintained, and thereby providing actionable practitioner guidance that the existing literature does not.

Fourth, COS introduces an ethics framework for neuroscience-informed organizational intervention. As neuroscience concepts become more prevalent in organizational consulting, the COS framework—grounded in autonomy, transparency, participation, and revocability—provides a model for responsible integration.

A potential challenge to COS’s novelty deserves engagement: does the framework offer genuine advance, or merely relabel existing constructs? COS brings together three moves not previously combined in this configuration: (1) a multi-level mechanism—the emergence bridge—connecting individual, interactional, and organizational levels into a single account; (2) operationalization into reproducible structural intervention techniques with explicit failure conditions; and (3) repositioning psychological safety from a climate variable to a structural attractor condition engineered through specific behavioral design. These generate different intervention designs and different testable predictions regarding sequencing, temporal thresholds, and failure conditions not derivable from the source frameworks independently.

The role of neuroscience in COS requires clarification. Neuroscience serves a specific function: providing theoretical inspiration for understanding behavioral self-sustenance at the individual level. The neuroscience of habit consolidation and repetition-based behavioral stabilization—how repetition reduces attentional resources required to initiate behavior (Kandel, 2006)—informs the design logic of Neural Base Design practices. Without this theoretical grounding, the emergence bridge would lack an account of why habitualized behaviors become self-maintaining. The neuroscientific layer does not imply reductionism; it provides theoretical coherence at the individual level within a multi-level framework, supporting the account of how behavioral habituation may aggregate into organizational-level dynamics.

### 6.2 Practical implications

Three implications for organizational development practitioners follow from the COS framework. First, *diagnostically*: before designing intervention, practitioners should seek to understand the current attractor state—the stable patterns of communication, decision-making, and interaction—rather than focusing immediately on desired behaviors. The question ‘What structural dynamics are reproducing this pattern?’ is prior to ‘What should we do to change it?’

Second, *sequencing*: the hierarchical architecture of COS techniques suggests that perturbation-focused interventions (Field Gradient Theory) and feedback redesign (Loop Conversion Design) are more likely to produce durable effects when preceded and accompanied by Neural Base Design’s foundational practices. Structural change attempted without relational foundation typically reverts.

Third, *time horizons*: attractor transition—as distinct from temporary behavioral change—requires a sustained period of consistent intervention, on the order of months, before autonomous self-sustenance is established. Practitioners and organizational leaders should calibrate expectations accordingly; the provisional six-month threshold proposed in Section 4.3 is a hypothesis for independent testing, not a guarantee.

### 6.3 Limitations

The most significant limitation is structural: the conceptual illustrations in Section 4 are not empirical observations and should not be interpreted as data derived from organizational engagements. They are theoretically derived archetypal patterns; as such, they create structural conditions for confirmation bias (Nickerson, 1998) in the sense that the same practitioners who designed the framework also selected which patterns to illustrate. The illustrations motivate hypotheses rather than confirm them. Future research should involve independent observation by researchers with no involvement in intervention design or delivery.

The neuroscientific elements operate as theoretical framework rather than direct neural measurement. Claims about specific mechanisms—social bonding systems, dopaminergic pathways, synaptic plasticity—are theoretical propositions awaiting empirical verification. The ecological validity of some sources (e.g., Algoe et al., 2010, from close-relationship contexts) in organizational settings requires verification in organizational samples. Finally, the evidence base is limited to Japanese organizational settings; generalizability across cultural contexts remains to be investigated.

### 6.4 Future research directions

The COS framework generates three specific testable propositions, each linked to plausible operationalization pathways within established measurement traditions. First, temporal: organizational attractor transition, measured by autonomous behavioral self-sustenance, occurs on the order of months following sustained Neural Base Design initiation, with approximately 6 months proposed as a provisional threshold—operationalizable through repeated-measures designs employing established psychological safety and voice behavior measures (e.g., Edmondson, 1999; LePine and Van Dyne, 1998) across organizational sites with independent assessment. Second, mechanistic: 3Good1More produces its effects through the cognitive broadening mechanism described by Fredrickson's (2001) Broaden-and-Build Theory—testable using validated measures of cognitive flexibility and attentional scope in pre-post designs. Third, structural: 2-on-1 interaction configurations produce more attractor-disrupting dynamics than 1-on-1 configurations—testable in experimental social psychology paradigms using interaction analysis methods. At a broader level, the framework invites collaboration between organizational psychology, implementation science, and complexity science researchers. Implementation evaluation frameworks such as PROLIFERATE (Pinero de Plaza et al., 2023) may offer complementary tools for assessing COS interventions in applied settings.

### 6.5 Boundary conditions

The COS framework is not equally applicable across all organizational contexts. Three boundary conditions warrant explicit statement. First, COS is most likely to produce durable attractor transition where leadership is genuinely committed to sustained structural change over a minimum of several months; organizations seeking rapid transformation are unlikely to reach the autonomous phase described in Section 4.3. Second, high power distance contexts—in which hierarchical authority is strongly internalized and challenge of senior members is experienced as illegitimate—present structural resistance to Field Gradient Theory’s perturbation mechanisms; the 2-on-1 configuration may be perceived as coercive regardless of Neural Base Design preparation. Third, organizations in acute crisis states are unlikely to sustain the attentional and relational resources Neural Base Design requires; in such contexts, crisis-oriented logics may temporarily supersede the preconditions COS requires. These boundary conditions specify the organizational terrain within which the framework is most likely to function as theorized.

## 7 Conclusion

Clinical Organizational Science reconceptualizes organizational transformation from a behavioral change project to a structural intervention problem. By integrating complexity science, neuroscience, organizational psychology, and behavioral science, COS specifies why organizations maintain stability—through the recursive reproduction of attractor states constituted by habitualized behavioral patterns aggregating through interaction into organizational routines—and how transformation occurs: through principled structural perturbation under conditions of psychological safety and ethical governance.

The three intervention techniques operationalize this framework in reproducible form, each targeting a distinct structural mechanism. Conceptual illustrations suggest patterns consistent with theoretical predictions; these illustrations motivate hypotheses rather than establish causal claims. COS claims principled probabilism—that by understanding the structural mechanisms of organizational stability and designing interventions accordingly, practitioners can increase the probability that transformation occurs and endures. In the complex domain, increasing the probability of favorable emergence is what science can offer.
